# Agent-Based Modeling of Autosomal Recessive Deafness 1A (DFNB1A) Prevalence with Regard to Intensity of Selection Pressure in Isolated Human Population

**DOI:** 10.3390/biology11020257

**Published:** 2022-02-07

**Authors:** Georgii P. Romanov, Anna A. Smirnova, Vladimir I. Zamyatin, Aleksey M. Mukhin, Fedor V. Kazantsev, Vera G. Pshennikova, Fedor M. Teryutin, Aisen V. Solovyev, Sardana A. Fedorova, Olga L. Posukh, Sergey A. Lashin, Nikolay A. Barashkov

**Affiliations:** 1 Laboratory of Molecular Biology, M. K. Ammosov North-Eastern Federal University, Kulakovskogo 46, 677010 Yakutsk, Russia; gpromanov@gmail.com (G.P.R.); rest26@mail.ru (F.M.T.); nelloann@mail.ru (A.V.S.); sardaanafedorova@mail.ru (S.A.F.); 2 Laboratory of Molecular Genetics, Yakut Science Centre of Complex Medical Problems, Yaroslavskogo 6/3, 677000 Yakutsk, Russia; psennikovavera@mail.ru; 3 Sector for Computer Analysis and Modeling of Biological Systems, Federal Research Center Institute of Cytology and Genetics, Siberian Branch of Russian Academy of Sciences, Lavrentyeva 10, 630090 Novosibirsk, Russia; asmirnova@bionet.nsc.ru (A.A.S.); zamyatin@bionet.nsc.ru (V.I.Z.); mukhin@bionet.nsc.ru (A.M.M.); Russia; kazfdr@bionet.nsc.ru (F.V.K.); lashin@bionet.nsc.ru (S.A.L.); 4 Novosibirsk State University, Pirogova 1, 630090 Novosibirsk, Russia; posukh@bionet.nsc.ru; 5 Laboratory of Human Molecular Genetics, Federal Research Center Institute of Cytology and Genetics, Siberian Branch of Russian Academy of Sciences, Lavrentyeva 10, 630090 Novosibirsk, Russia

**Keywords:** agent-based computer modeling, hereditary deafness, *GJB2*, genetic fitness, assortative mating, sign language, isolated population

## Abstract

**Simple Summary:**

In this study, we developed a simple simulation model to illustrate the effects of different mating patterns on the spread of autosomal recessive deafness 1A (DFNB1A) in an isolated human population with regard to the intensity of selection pressure. The modeling results have revealed that the prevalence of DFNB1A in an isolated population can be dramatically increased under frequent assortative marriages in a relatively short time period under the pressure of “relaxed” selection. However, under current conditions, the proportion of recessive homozygotes quickly reaches a short plateau and then continuously decreases. Moreover, in the long term, the studied effect can be leveled by growing social equality for deaf people, as evidenced by the results of neutral selection modeling.

**Abstract:**

An increase in the prevalence of autosomal recessive deafness 1A (DFNB1A) in populations of European descent was shown to be promoted by assortative marriages among deaf people. Assortative marriages became possible with the widespread introduction of sign language, resulting in increased genetic fitness of deaf individuals and, thereby, relaxing selection against deafness. However, the effect of this phenomenon was not previously studied in populations with different genetic structures. We developed an agent-based computer model for the analysis of the spread of DFNB1A. Using this model, we tested the impact of different intensities of selection pressure against deafness in an isolated human population over 400 years. Modeling of the “purifying” selection pressure on deafness (“No deaf mating” scenario) resulted in a decrease in the proportion of deaf individuals and the pathogenic allele frequency. Modeling of the “relaxed” selection (“Assortative mating” scenario) resulted in an increase in the proportion of deaf individuals in the first four generations, which then quickly plateaued with a subsequent decline and a decrease in the pathogenic allele frequency. The results of neutral selection pressure modeling (“Random mating” scenario) showed no significant changes in the proportion of deaf individuals or the pathogenic allele frequency after 400 years.

## 1. Introduction

Hearing loss (HL), caused by both environmental and genetic factors, affects more than 10% of the world’s population and is associated with disability and significantly reduced quality of life among affected individuals. On average, 1 in 1000 newborns is born deaf and, in 50–60% of cases, the pathology has a genetic cause [1,2]. Hereditary HL cases are subdivided into two forms: non-syndromic (isolated HL) and syndromic (HL in addition to other clinical traits). Syndromic HL comprises roughly 30% of all HL cases, with more than 400 HL-associated syndromes having been described, while the remaining 70% are non-syndromic [3]. Hereditary non-syndromic HL is a monogenic disease with uniquely high genetic heterogeneity. Around 160 genetic loci associated with non-syndromic HL are currently known, and about 120 genes have been identified, mutations that led to hearing impairment [4]. Autosomal recessive deafness 1A (DFNB1A), caused by mutations in the *GJB2* gene (MIM 121011, 13q12.11) encoding the protein connexin 26 (Cx26), is the most prevalent in many populations [5]. The proportion of DFNB1A among hereditary forms of HL is 17.3% worldwide and reaches up to 27.1% in populations of European descent [5]. In total, about 400 mutations in the *GJB2* gene are known, the majority of which are recessively inherited [6]. A varying prevalence of different *GJB2* mutations has been shown in various populations worldwide, which can be explained by large-scale populational events throughout history [7,8,9,10,11,12,13]. The unique *GJB2* mutational spectrum and the accumulation of certain *GJB2* mutations in certain ethnic groups can be attributed to the founder effect [14,15,16,17,18,19,20,21,22].

At the same time, Nance et al. [23,24] suggested that certain social factors could be a strong driving force behind the increased incidence of DFNB1A in developed countries, due to relaxed selection against deafness. This phenomenon began after the introduction of sign language 400 years ago in many Western countries and the subsequent establishment of residential schools for the deaf [23,24]. It was hypothesized that sign language-based homogamy among deaf people promoted assortative marriages between them and consequently improved genetic fitness (reproductive capabilities). This hypothesis was later supported by a comparative analysis of modern and retrospective demographic parameters of the deaf population in the USA [25,26]. Thus, it was evidenced that the combined effect of a high assortative mating rate and increased genetic fitness of deaf people may have doubled the frequency of DFNB1A in the United States over the past 200 years [23,25]. The impact of assortative mating by deafness on the incidence of DFNB1A is defined by the proportion of marriages in which both partners are homozygous by recessive pathogenic allele and hence have a possibility of having only deaf children; such marriages are termed as non-complementary [23,24] (Supplementary Materials, Chapter S1). On the other hand, complementary marriages are expected to have hearing children since each partner has a different etiology of HL.

However, using computer simulations, it was shown that this effect is limited only to the most frequent recessive form of hereditary HL, and the influence on the total prevalence of deafness in the population was found to be insignificant due to the high heterogeneity of HL, both hereditary and nonhereditary etiology [27]. These results are in accordance with theoretical models of non-random mating. Assortative mating was originally mathematically studied by R.A. Fisher [28] and S. Wright [29] and later reworked by Crow and Felsenstein [30]. Their general conclusion was that in the case of recessive trait assortative mating, recessive homozygosity increases, and most of the increase occurs in the first generations, while the underlying allelic frequencies in the population do not change. While it is evident that the emergence of assortative mating changed the pressure of natural selection on recessive deafness in relatively large populations of European descent, it is unclear what level of impact it will have on populations with different genetic backgrounds and social structures. Thus, in this study, we present an analysis of computer modeling of autosomal recessive deafness 1A (DFNB1A) prevalence in an isolated human population under different evolutionary scenarios.

## 2. Materials and Methods

Agent-based modeling can simulate very specific behaviors of individuals depending on the environment and is a widely used tool for investigating the spreading of human diseases [31,32,33,34,35]. It is utilized to model infectious diseases like HIV [36,37,38], COVID-19 [39] and others [40,41], as well as non-infectious diseases like diabetes mellitus [42,43], obesity [44], Alzheimer’s disease [45]. To assess the prevalence of congenital recessive HL under different intensities of selection pressure for deafness in an isolated human population, we developed an agent-based model that simulated a phenotype-based mating behavior. For the purposes of the correct modeling of the spread of DFNB1A, an isolated population with known data on the prevalence of *GJB2* gene mutations causing hereditary HL and data on the proportion of assortative mating among deaf people and their reproductive capabilities in comparison with their hearing siblings were needed.

### 2.1. Reference Population

Data from the Yakut population was used as a reference for the developed model. The Yakuts (originally known as the Sakha) are the largest population of indigenous people in Siberia (466,492 according to the Russian Census, 2010) living in the Sakha Republic (Eastern Siberia, Russia). The Yakuts are characterized by specific anthropological, demographic, linguistic and historical features linked to their relationships with the nomadic Turkic tribes of South Siberia and Central Asia. The genetic data revealed a relatively small size of the Yakut ancestor population and a strong bottleneck effect in the Yakut paternal lineages (around 80% of the Y chromosomes of the Yakuts belong to one haplogroup, N3) [46]. Marriage traditions and geographical isolation played a significant role in the genetic and demographic history of the Yakut population. A high frequency of some Mendelian disorders in the Yakut population was found to be the result of the founder effect. For example, the high prevalence of HL in Yakuts is caused by the founder c. − 23 + 1 G > A mutation in the *GJB2* gene (92.2% of all mutant *GJB2* alleles found in deaf patients), which was found with extremely high carrier frequency among hearing Yakut individuals (10.3% of the total population) [17,47]. Moreover, the data on marriage structure and reproduction of deaf people living in the Sakha Republic were presented in comparison with the contribution of *GJB2* gene mutations to the etiology of HL. The relative fertility of deaf people compared with their hearing siblings was 0.78 (mean number of children 1.76 and 2.24 for deaf individuals and their hearing siblings, respectively) [48]. The rate of assortative marriages among deaf people was 77.1% [48]. The known genetic structure of hereditary HL in Yakuts and the availability of data on the reproductive capabilities and marital structure of deaf people make this population suitable for computer simulation of the distribution of DFNB1A.

### 2.2. The Model

To build our model, we used the C++ programming language and the Microsoft Visual Studio 2019 (ver 16.8.4) development environment. The key element of the model is a decentralized agent, which represents an individual with a set of parameters defining their mating behavior. The main cycle of the algorithm represents one generation (one generation = 20 years). The generations in the model are non-overlapping.

At the start, the program generates agents according to the initial size of the population and the proportions of phenotypes and genotypes. A genotype is represented by two independent alleles. Each allele is assigned the values “1” or “0”, where 1 is the recessive mutant allele (deafness allele), and 0 is the dominant normal allele. Thus, recessive homozygotes are deaf. Additionally, the agent will be deaf regardless of genotype according to the non-genetic deafness proportion value specified in the initial parameters. Whether the agent will know sign language is defined according to the proportion value in the initial parameters, which can be specified for deaf and hearing agents. Thus, each agent in the model has the following parameters:

- sex—male or female; main criterion in marriage step of model algorithm;
- genotype with two alleles—each allele can be mutant or normal;
- phenotype—true if agent is deaf, false if agent is hearing;
- sign language—knowledge of sign language (true/false).

**Figure 1:**
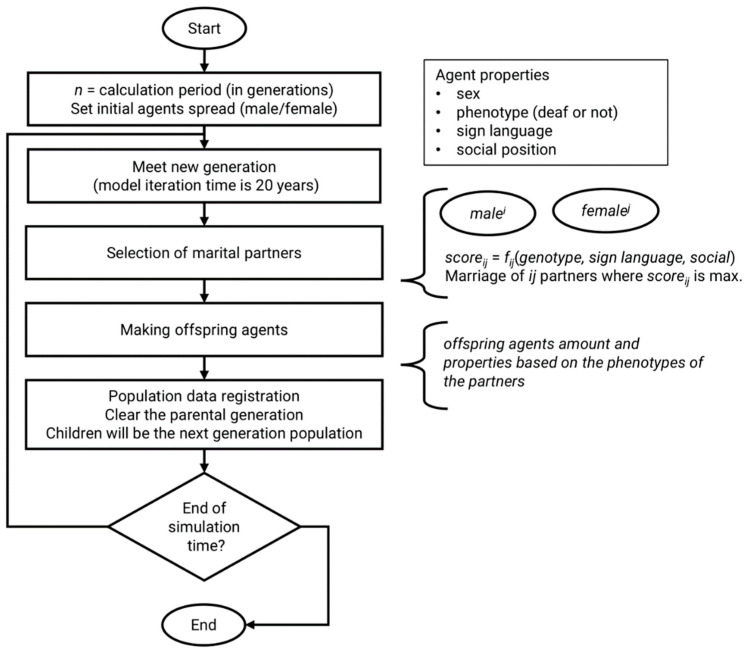
Simplified scheme of the main cycle of the program. At the initial stage, a population of agents (individuals) is created according to the parameters set by the user. The population of the next generation consists of the progeny of the agents of the previous generation.

After the creation of the population, the main process starts with the formation of couples. To simulate the process of agent marriage, a matrix is formed, the first row of which corresponds to male agents ID and the first column to female agents ID. Each cell of this matrix contains an “S” score assigned to each potential couple according to the algorithm for the mutual evaluation of potential partners (Equation (1)). A simplified scheme of the main cycle of the program is demonstrated in Figure 1.

The algorithm for the mutual evaluation of candidate agents calculates the “*S*” score for a potential couple based on the similarity of their phenotypes and their knowledge of sign language. Preference for similarity of these parameters by agents can be regulated in order to meet the criteria of assortative mating, which is implemented by assigning “weights” to the parameters of phenotype and sign language for all agents. The variables *weight_pheno_h* and *weight_pheno_d* define the value of similarity of their phenotype for candidate agents, hearing and deaf, respectively. For brevity, in the manuscript, we further refer to these variables as *W^H^_P_* (*weight_pheno_h*) and *W^D^_P_* (*weight_pheno_d*). The variables *weight_sign_h* and *weight_sign_d* define the value of knowledge of sign language for candidate agents, hearing and deaf, respectively, hereinafter referred to as *W^H^_S_* (*weight_sign_h*) and *W^D^_S_* (*weight_sign_d*). This allowed us to regulate whether these parameters will equally determine the attractiveness of a potential partner, or one of them will make up a larger part of the resulting “*S*” score. If both candidates are hearing, the values of the *W^H^_P_* parameters of both candidates are added to the “*S*” score (i.e., 2 × *W^H^_P_*). If both candidates are deaf, the value 2 × *W^D^_P_* is added to the “*S*” score, and 2 × *W^D^_S_* is added if both candidates know sign language (Equation (1)). If one candidate agent is deaf and the other is hearing, the sum (*W^H^_S_ + W^D^_S_*) will be added to the “*S*” score only if both candidates know sign language. Thus, agents with similar phenotypic parameters will have higher scores.

(1) $$S_{ij}\left(male_{i},\;female_{j}\right)\;=\{\begin{array}{c} \begin{array}{c} 2\times W_{P}^{H},\mathrm{if}\;\mathrm{mal}e_{i}.\mathrm{phenotype}=\mathrm{femal}e_{j}.\mathrm{phenotype}=\mathrm{false}\\ 2\times W_{P}^{D},\mathrm{if}\;\mathrm{mal}e_{i}.\mathrm{phenotype}=\mathrm{femal}e_{j}.\mathrm{phenotype}=\mathrm{true}\\ 2\times (W_{P}^{D}+W_{S}^{D})\;,\mathrm{if}\;\mathrm{mal}e_{i}.\mathrm{phenotype}=\mathrm{femal}e_{j}.\mathrm{phenotype}=\mathrm{true}\;\mathrm{and}\;\mathrm{both}\;\mathrm{signlanguage}=\mathrm{true} \end{array}\\ W_{P}^{D}+W_{S}^{D}\;,\mathrm{if}\;(\mathrm{mal}e_{i}.\mathrm{genotype}!=\mathrm{femal}e_{j}.\mathrm{phenotype})\;\mathrm{and}\;\mathrm{both}\;\mathrm{signLanguage}=\mathrm{true}\\ 0,\;in\;other\;ways \end{array}$$

Next, if the resulting “*S*” score is greater than the lower threshold values of both candidate agents, which are defined by the “*socialPosition*” parameter, then the value of “*S*” is recorded in the cell of the matrix corresponding to a given pair of candidates (Equation (2)).

(2) $$M_{ij}=\;\{\begin{array}{c} S\left({\mathrm{male}}_{i},{\;\mathrm{female}}_{j}\right),\;if\;S\left({\mathrm{male}}_{i},{\;\mathrm{female}}_{j}\right)>\max\left(min\left({\mathrm{male}}_{i}.\mathrm{socialPosition},{\;\mathrm{female}}_{j}.\mathrm{socialPosition}\right)\right)\;\\ 0,\;in\;other\;way \end{array}$$

Next, the program selects a pair of candidate agents with the highest score in the *M* matrix, and a couple is created. If there are two or more pairs with equally high scores, one of them is selected randomly. The selected agents become unavailable to other candidate agents. We must also note that a pair of candidate agents with a low “*S*” score will also have a chance of “marriage” if it is higher than the “*socialPosition*” value and there are no higher scores. After all of the couples are formed, the process of offspring generation starts.

The offspring generation algorithm considers the phenotypes of the partners (DD, DH or HH, where D is deaf and H is hearing) and a mean number of children (birthrate), which is specified in the initial parameters for each type of couple. For every couple, the number of children is defined individually using generated values corrected with a beta distribution. The program creates the corresponding number of new agents, which are equally likely to be male or female. The parental genes are inherited by a new agent with equal probability; there are no de novo pathogenic alleles. The possibility of non-genetic deafness is determined in the same way as described above for the initial population. After offspring are generated for every couple, the parental agent population is deleted and replaced by the new agents, and the process of couple formation starts again. A detailed description of the algorithm and a list of the input parameters are presented in the Supplementary Materials, Chapters S2 and S3.

For the batch operation of the program and statistical processing of the output data, we developed a service script that controls the number of simulation runs, sets the starting parameters for the model, performs statistical calculations and generates summary plots. The script was written in the Python programming language using the pandas and matplotlib libraries. A single simulation of the “Assortative mating” scenario takes approximately 70 s on 1 thread of the 3.5 GHz AMD Threadripper 1920X processor (Advanced Micro Devices, Inc., Santa Clara, California, USA) with an SSD hard drive. The program can be run on both Windows and Linux operating systems.

### 2.3. Verification and Validation of the Model

**Figure 2:**
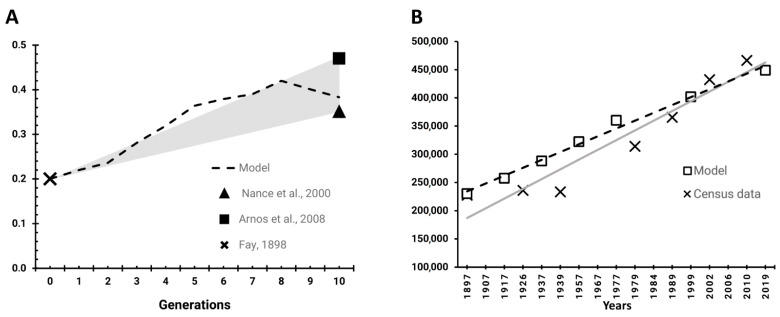
Model validation and verification. (**A**) Comparison of model outcome with archive data for the USA deaf population. Increase in the proportion of recessive mutant homozygotes in the model outcome (dotted line with diamonds—10 data points) was comparable to the data reported by Nance et al. [23] and Arnos et al. [25]. (**B**) Comparison of model outcome with archive census data on the Yakut population. The trend of population size increase in the model outcome (black dashed line with squares) was comparable to the actual growth of the Yakut population according to census data (grey solid line with crosses).

The correct implementation of the sign language-based assortative mating mechanism in our model was verified by reproducing the outcomes of previous studies on the analysis of linguistic homogamy’s influence on recessive hereditary deafness in the USA [23,25] (Figure 2A; Supplementary Materials, Chapter S4). The model was run using data from the nationwide sample of pedigrees of deaf marriages between 1803 and 1894 [49] in order to reach the modern characteristics of the deaf population in the USA [23,25]. The current proportion of people with autosomal recessive deafness among the deaf population in the USA was estimated to be 0.35 [23] and 0.47 [25], which is 1.5 to 2.4 times higher than that of the 19th century (0.2) [49].

The model parameters were set to meet the reported characteristics [23,25,49], and the initial prevalence of individuals homozygous for *GJB2* gene mutations was set at 0.2%. The proportion of assortative marriages among deaf individuals was set to 76%; the marriage rate and fitness of hearing and deaf individuals were equal, with a mean number of 2.1 children per marriage; deaf individuals were assumed to know sign language; the modeling time was set to 10 generations (200 years), the model was run 1000 times; and 95% confidence intervals were calculated. Modeling under these parameters indicated that the proportion of deaf homozygotes increased 1.9 times (from 0.2% to 0.38%) over 200 years, which corresponds to the 1.5–2.4 times increase reported in the USA. Thus, the modeled dynamics of recessive mutant homozygotes in the model outcome were in accordance with the data reported by Nance et al. [23] and Arnos et al. [25] (Figure 2A).

In order to verify the model on the reference Yakut population, we reproduced the population growth according to archive and modern census data, without consideration of pathogenic allele frequency and mating characteristics. According to the census data, the Yakut population was 227,384 in 1897 and had increased to 466,492 by 2010. The birth rate value was set to 2.24, which is a mean value for the number of children who survived to reproductive age in 1897 (2.18 per woman) and 2010 (2.31 per woman), according to archive census data. By assigning these values in the initial population number and birthrate parameters, after 100 years (five generations), the population growth in the model outcome was comparable to the census data for the same period (Figure 2B; Supplementary Materials, Chapter S4).

### 2.4. Simulation Scenarios

We carried out the simulations with three different combinations of initial parameters for the model population (Supplementary Materials Table S1). In the first scenario, the agents (individuals) did not know sign language, and there was no deaf community (“No deaf mating” scenario). In the second scenario, deaf individuals used sign language and formed a community (“Assortative mating” scenario). In the third scenario, all agents were mating regardless of their phenotype (“Random mating” scenario). Other parameters were set in order to meet actual data from the Yakut population. The initial frequency of recessive homozygous agents was set to 0.25%, which is calculated from the registered frequency of heterozygous individuals (10.3%) [17]. The proportion of non-genetic deafness was also set to 0.25% in accordance with the DFNB1A contribution in the etiology of HL in the Yakut population [17]. The average number of children in the marriage of two deaf agents was set to 1.78; of deaf and hearing was set to 1.7; and of two hearing agents was set to 2.24 children, in order to represent registered reduced relative fertility (by 22%) of deaf people in Yakutia [48]. The assortative mating rate was set to 77.1% [48]. To generate reliable statistical data, the simulation of each scenario was performed 1000 times. Statistical processing was carried out by calculating 99% confidence intervals for each set of values (*n* = 1000) of the variables produced by the program.

## 3. Results

We developed an agent-based computer model for analysis of the spread of hereditary congenital recessive HL in an isolated human population (Supplementary Materials, Chapter S5). The agent in this model was a single decentralized individual. Each agent was characterized by their phenotype and genotype. The main phenotypic parameters were: sex (male/female), hearing status (deaf/hearing) and sign language (knowledge/ignorance). The genotype was represented by one locus/gene in which a recessive allele is pathogenic. The main algorithm of the program represents the life cycle of one generation (which was considered to be 20 years) of the model population. One cycle of the program includes the choice of marital partners based on phenotype, creation of a new population consisting of the progeny of agents of the current generation and modeling of consolidated communities of deaf people. We ran the model in three different scenarios (different combinations of initial parameters for the model population) in order to simulate changes in DFNB1A prevalence under different intensities of selection pressure. For each generation, the program registered data on the total population number and the number of deaf individuals, calculated the proportion of recessive mutant homozygotes and the frequency of recessive mutant alleles and compiled these parameters into tables.

### 3.1. The Scenario “No Deaf Mating”

**Figure 3:**
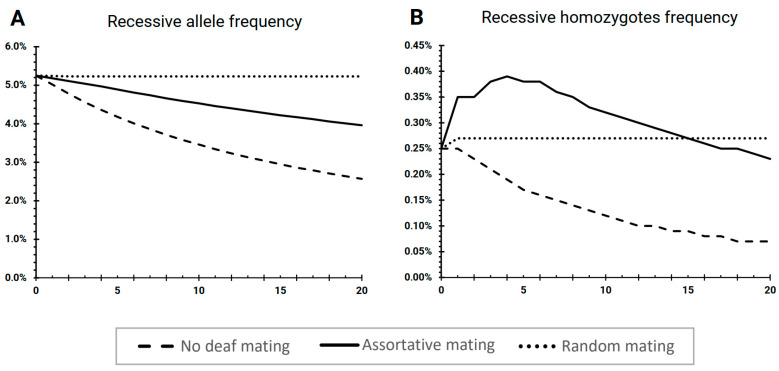
Model outcomes for different scenarios. (**A**) Frequency of recessive mutant allele. (**B)** Proportion of deaf individuals (recessive mutant homozygotes). Y-axis: proportion, X-axis: generations (1 generation = 20 years).

The first scenario, “No deaf mating”, was a model of a population where deaf individuals did not mate and had no progeny, hence representing the full pressure of “purifying” selection against deafness. The simulation results revealed an increase in the population size from the initial 200,000 to 1,721,203.74 (99% CI = ± 29.72) individuals by the 20th generation, and the number of deaf individuals increased from an initial 999.31 (± 0.37) to 5440.07 (± 14.01). The frequency of the recessive mutant allele decreased from 5.25% to (± 0.00) to 2.57% (± 0.00) (Figure 3A) after 20 generations. The proportion of deaf individuals (recessive mutant homozygotes) continuously decreased from 0.25% (± 0.00) to 0.07% (± 0.00) by the 20th generation (Figure 3B).

### 3.2. The Scenario “Assortative Mating”

The second scenario, “Assortative mating”, was a model of a population with the presence of sign language, and 77.1% of marriages of deaf people were assortative. This scenario represented “relaxed” selection due to the presence of linguistic homogamy among deaf individuals. The simulation results revealed that the population number increased from an initial 200,000 to 1,814,625.89 (± 142.06) individuals by the 20th generation, and the number of deaf individuals increased from an initial 997.60 (± 1.55) to 8,719.41 (± 15.80). The frequency of the recessive mutant allele decreased from 5.25% (± 0.00) to 3.96% (± 0.00) after 20 generations (Figure 3A). The proportion of recessive mutant homozygotes increased up until the 4th generation, from 0.25% (± 0.00) to 0.39% (± 0.00), and then decreased to 0.23% (± 0.00) by the 20th generation (Figure 3B).

### 3.3. The Scenario “Random Mating”

The third scenario, “Random mating”, was a model of a population where all individuals mated randomly regardless of their phenotypes. This scenario represented neutral selection due to a seeming lack of pressure on the deafness phenotype. In this scenario, the population number increased from an initial 200,000 to 1,868,655.58 (± 345.94) individuals by the 20th generation, and the number of deaf individuals increased from an initial 999.0 (± 0.81) to 9776.90 (± 0.16). The frequency of the recessive mutant allele slightly lowered in the first two generations from an initial 5.25 (± 0.00) to 5.23% (± 0.00) and then remained constant during 18 generations of modeling (Figure 3A). The proportion of recessive mutant homozygotes increased slightly from 0.25% to 0.27% in the 1st generation and then also remained constant until the 20th generation (Figure 3B). The total population number dynamics between all three scenarios were comparable. The number and proportion of deaf individuals (recessive mutant homozygotes) and the frequency of recessive alleles changed variably in each scenario, depending on the intensity of the modeled selection pressure.

## 4. Discussion

In this study, we developed a simple model to illustrate the effects of different intensities of natural selection on the spread of autosomal recessive deafness in an isolated human population. In contrast to previously presented models [24,27], the agent-based model developed in this study explicitly describes the sign language appearance in deaf communities and proposes an algorithm for the selection of marital partners based on preference for certain phenotypic parameters. In order to test the different levels of selection pressure on deafness, we ran the program under three different scenarios (different sets of initial parameter combinations for the model population).

The model population of the “No deaf mating” scenario resulted in a decrease in the proportion of deaf individuals (from 0.25% to 0.07%) and the frequency of the pathogenic allele (from 5.25% to 2.57%) (Figure 3). This scenario assumed that deaf people could not marry unless they used sign language to communicate (linguistic homogamy) and therefore could not have offspring without it. Thus, the genetic fitness of deaf individuals was close to zero, which represented a high selection pressure against deafness. According to this scenario, deaf children could be born (with a 25% probability) only from hearing parents who were both heterozygous carriers of a recessive pathogenic allele. Consequently, the observed continuous decrease in mutant allele frequency was the result of a decreasing proportion of recessive mutant homozygotes in the population. Currently, the indigenous population of Yakutia (Eastern Siberia, Russia) is characterized by a relatively recent establishment of schools for deaf and hard of hearing and the highest heterozygous carrier rate of DFNB1A causing mutation due to strong founder effect and genetic isolation. Thus, this scenario represents a hypothetical outcome in the Yakut population, where the genetic fitness of deaf individuals is extremely low due to the absence of a consolidated community based on specialized education. Such a situation was virtually possible in Yakutia up until the emergence of the first school for deaf and hard of hearing in 1951.

The model population of the “Assortative mating” scenario showed that the proportion of recessive homozygotes increased 1.5 times (from 0.25% to 0.39%) in the first four generations (80 years), and then decreased to 0.23% by the 20th generation (400 years) (Figure 3B). The frequency of recessive mutant alleles decreased from 5.25% to 3.96% (Figure 3A). These data suggest that in a population with high heterozygosity (10.3%), assortative marriages between deaf people can increase the initial incidence of hereditary HL (Figure 3B), as was previously shown in other studies of the potential influence of social factors on hereditary HL [24,25,26]. A following decrease in the proportion of recessive mutant homozygotes after the 4th generation was associated with the 22% reduced fertility of deaf individuals relative to hearing individuals [48]. Thus, we can assume that if current reproductive parameters (0.78 relative fertility) and marital structures (77.1% of assortative mating) of deaf people in Yakutia remain unchanged, we expect up to a 1.5 times increase in DFNB1A cases in the next 80 years, which then will be followed by a prolonged decrease. However, if the relative fertility of deaf people increases, the incidence of recessive HL could possibly reach a new equilibrium level.

Modeling of the “Random mating” population indicated that the prevalence of hereditary HL did not change, in contrast to the “Assortative mating” (Figure 3). In this case, there were no assortative marriages by deafness since all individuals mate randomly, and the genetic fitness of all individuals was considered equal, regardless of their genotype. Therefore, the proportion of recessive homozygotes (*q*^2^) in the population will determine the probability of marriage between two deaf individuals (*q*^2^ × *q*^2^), and the proportion of such marriages will be much lower than in a population with assortative mating by deafness. This scenario represents a panmictic population in which all individuals have equal genetic fitness, and the proportions of genotypes and allele frequencies will remain constant from generation to generation according to the Hardy–Weinberg principle. A similar “panmictic” scenario was tested in two previous studies [24,27]. Nance and Kearsey modeled a population with a totally random choice of a partner (random mating) and equal reproductive capabilities of deaf and hearing individuals, which resulted in a minimal increase in the proportion of mutant homozygotes (by around 1.5%) over 400 years [24]. Braun et al. demonstrated that in a model population with random mating, the frequency of the pathogenic allele and the proportion of mutant homozygotes did not change over 200 years (10 generations) [27]. Such a scenario could be possible in the near future when all people in a population will have equal social accessibility, which could be provided by massive availability of communicational and informational resources, education and healthcare.

Several study limitations and modeling assumptions may have affected our results. First, while we were confident in the current parameters of relative fitness, mating rate and prevalence of *GJB2* gene causative variants of deaf individuals in Yakutia [48], it is unknown how these parameters could change in the near future. Further, we did not include other hereditary forms of HL in the model, and their contribution to the structure of deafness in the Yakut population has not been explicitly defined. More realistic and complex agent-based simulations including these factors would provide a better understanding of their interactions and support more solid predictions. Despite these limitations, the results of this study emphasize how agent-based computer simulations provide a powerful tool for the analysis of autosomal recessive deafness dynamics in isolated human populations under different mating patterns.

Modeling and predicting the dynamics of hereditary deafness is complicated by the high heterogeneity of HL. More causative genetic loci (two or even three) occurring in different populations of interest need to be considered with regards to mating structure. Moreover, translation of the model to a different scale would reveal previously unconsidered issues. Modeling of large populations, e.g., metropolises, cannot be simply implemented by increasing the number of agents in a simulated population. To achieve this, data on the interactions of large groups of people are needed. We assumed that interactions in very large populations (tens or hundreds of millions of people) are, in fact, interactions of practically independent communities of a smaller scale (from tens of thousands to a million). In this regard, this study was devoted to such relatively small communities. After we obtain a clearer understanding of how the interactions within them function, it will be possible to study larger communities.

## 5. Conclusions

In this study, we developed a simple simulation agent-based model to illustrate the effects of different mating patterns on the spread of autosomal recessive deafness in an isolated human population with regard to the intensity of selection pressure. The modeling results of the purifying” selection pressure on deafness (“No deaf mating” scenario) resulted in a decrease in the proportion of deaf individuals and the frequency of the pathogenic allele. The modeling results of “relaxed” selection (“Assortative mating” scenario) have revealed that prevalence of DFNB1A in an isolated human population can be dramatically increased under frequent assortative marriages in the relatively short time period. However, under current conditions, the proportion of recessive homozygotes quickly reaches a short plateau and then continuously decreases. Moreover, in the long term, the studied effect can be leveled by growing social equality for deaf people, as evidenced by the results of neutral selection modeling (“Random mating” scenario).

## Data Availability

The source code and all the experimental data are available at the following link: https://drive.google.com/drive/folders/1XHV9m-onPyWj8R-bX1fE1_J7XODoWbpT?usp=sharing (accessed on 17 December 2021).

## Supplementary Materials

The following supporting information can be downloaded at: https://www.mdpi.com/article/10.3390/biology11020257/s1, Figure S1. Combinations of marital partners by phenotype and genotype and possible genotypes in children; Figure S2. The simplified visualization of mutual assessment of candidate partners in case of the “assortative mating” scenario; Figure S3. Model verification on Yakut population size dynamics. A. Yakut population number according to archive census data. B. Population size increase in the model outcome; Birth rate values was set to 2.24, which is a mean value for the number of children who survived to reproductive age in 1897 (2.18 per woman) and 2010 (2.31 per woman), according to archive census data; Figure S4. Simulation results for scenario 1—“No deaf mating”. A—Total population size. B—The number of deaf individuals. C—Proportion of recessive mutant homozygotes in total population. D—Frequency of recessive mutant allele in total population; Figure S5. Simulation results for scenario 2—“Assortative mating”. A—Total population size. B—The number of deaf individuals. C—Proportion of recessive mutant homozygotes in total population. D—Frequency of recessive mutant allele in total population; Figure S6. Simulation results for scenario 3 “Random mating”. A—Total population size. B—The number of deaf individuals. C—Proportion of recessive mutant homozygotes in total population. D—Frequency of recessive mutant allele in total population. Table S1. Initial simulation parameters for different scenarios; Table S2. Simulation results for validation scenario by USA data; Table S3. Simulation results for scenario 1 “No deaf mating”; Table S4. Simulation results for scenario 2 “Assortative mating”; Table S5. Simulation results for scenario 3 “Random mating”.
