# Supplementary material for: Agent-Based Modeling of Autosomal Recessive Deafness 1A (DFNB1A) Prevalence with Regard to Intensity of Selection Pressure in Isolated Human Population
Source: Biology (Basel). 2022 Feb 7;11(2):257. doi: 10.3390/biology11020257 (PMC8869167; doi:10.3390/biology11020257)
Supplement: Supplementary file 1 [file biology-11-00257-s001.zip › biology-1542243-supplementary.pdf]

Supplementary Materials

- Chapter S1. Combinations of phenotypes and genotypes of marital partners ____________1
- Chapter S2. Program algorithm____________3
- Chapter S3. Initial parameters____________6
- Chapter S4. Verification and validation of the model____________8
- Chapter S5. Modeling results____________9

Chapter S1. Combinations of phenotypes and genotypes of marital partners

Possible combinations of marital partners according to their phenotypes are presented in the scheme (Figure S1). In the absence of sign language, deaf individuals practically do not marry and, as can be seen from the scheme, a child with hereditary HL can only be born to a couple of hearing individuals who are heterozygous carriers of a recessive mutant allele. With the emergence of sign language, the marriages among the deaf become possible; the likelihood for them to have deaf children varies in different combinations of married couples with genetic and non-genetic causes of deafness.

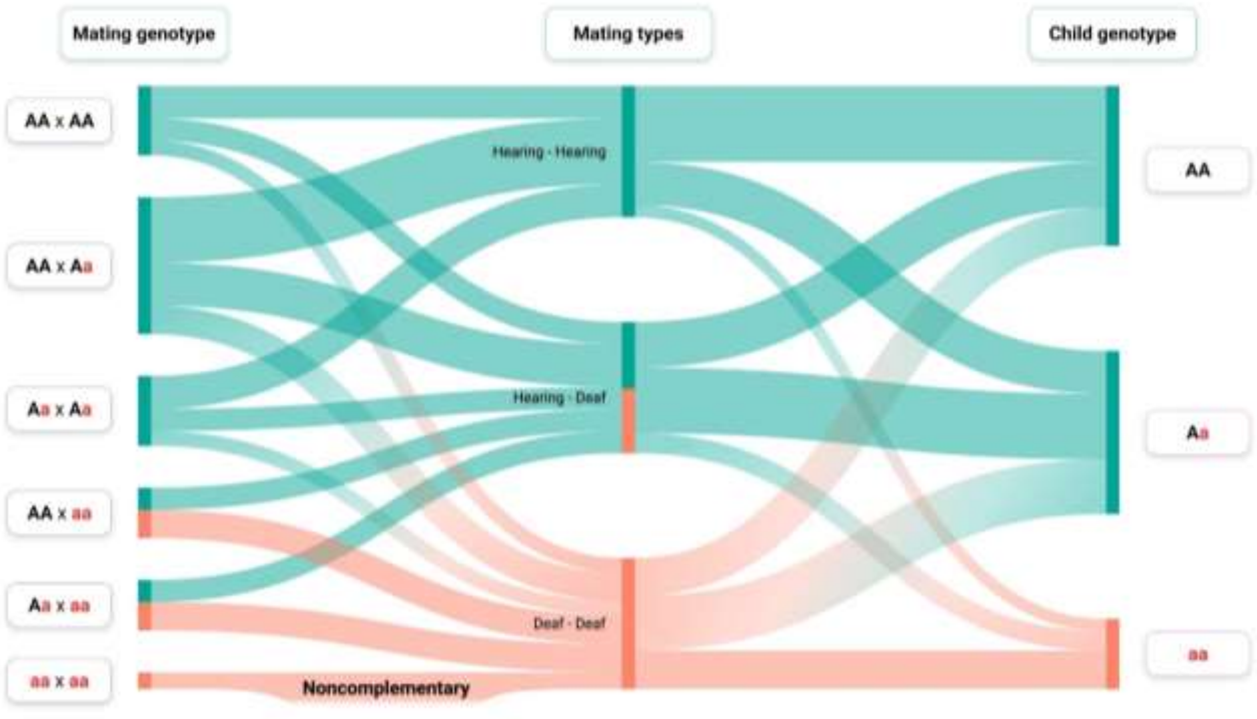

Figure S1. Combinations of marital partners by phenotype and genotype and possible genotypes in children. On the left are genotypes of marital partners. In the middle are the marriages types by phenotypes of partners. On the right are possible genotypes in children of different marriages types. A – normal allele. a – mutant recessive allele.

An analysis of assortative marriages of deaf individuals, taking into account the ability to hear of their descendants, makes it possible to divide them into two groups - the so-called complementary

and non-complementary assortative marriages [23]. Complementary marriages mean the marriages between deaf marital partners with different etiology of HL (acquired HL in one of the partners or mutations in different genes associated with HL); in such marriages, there may be only hearing or, in some cases, both deaf and hearing children. Non-complementary marriages are the marriages between deaf individuals who have the same genetic cause of HL – the presence of biallelic recessive mutations of the same gene. All children of such married couple will also be deaf and have the same genetic etiology of HL as their parents. From this category of marriages, the transmission of mutant alleles to the next generation is expected to be much higher than on average [24, 25]. The introduction of cochlear implantation leads to the emergence of new combinations of marital partners, both by phenotype and genotype (Figure S1).

Chapter S2. Program algorithm

At the first iteration, the program generates the initial population which consists of the arrays "men" and "women" filled by the agents according to the specified numbers of men and women (parameters INIT_POP_SIZE_M and INIT_POP_SIZE_W). When each agent is generated, his/her genotype is specified using the parameters alleleFather and alleleMother which can take on the values 0 or 1, where 1 is the recessive mutant allele (deafness allele), and 0 is the dominant normal allele. Proportion of genotypes that we set in initial parameters determines number of individuals with according values of alleleFather and alleleMother parameters.

To determine whether new agent will have deafness phenotype, the sum of the values of the alleleFather and alleleMother parameters is determined and if it is greater than 1, then the agent is considered to be deaf (the value of the isDeaf parameter is set to true), otherwise the agent is considered to be hearing (the value of the isDeaf parameter is set to false). Then the program specifies whether the agent will have acquired (non-hereditary) deafness similar to the setting of the previous parameters - if a random number from a uniform distribution is less than the value of the SPONTANEOUS_DEAF parameter that we specified, then the agent will be deaf, regardless of genotype.

Next, we specify the value of the agent's socialPosition parameter which is the minimum required sum of the values of the estimated parameters of the partner for the possibility of marriage. The value of the socialPosition parameter is set as a random number from the normal distribution with the mean equal to the SOCIAL_MEAN_D parameter and the standard deviation equal to SOCIAL_VAR_D if the agent is deaf. If the agent is hearing, then the mean and standard deviation are equal to SOCIAL_MEAN_H and SOCIAL_VAR_H, respectively. Whether the agent will know the sign language is specified by a random value from a uniform distribution if it is less than value of sign_lang parameter.

Starting from the 2nd iteration, the new population consists only of offspring from the previous iteration. All parameters of the agent described above are specified in a different way, described in the section "Algorithm for generating offspring". After the creation of the population, the main process starts - the formation of couples and the birth of children.

Algorithm for mutual assessment of partners for marriage

To simulate the process of marriage of agents, a matrix is formed, the first row of which correspond to male agents ID and the first column to female agents ID (Figure S2). Each cell of this matrix contains the so-called "score" s assigned to each potential couple according to the algorithm for mutual evaluation of partners. Next, line by line, for each male agent the program selects a female agent

with the highest "score"; a couple is created and the offspring is generated, after which the selected female agent becomes inaccessible to other candidates.

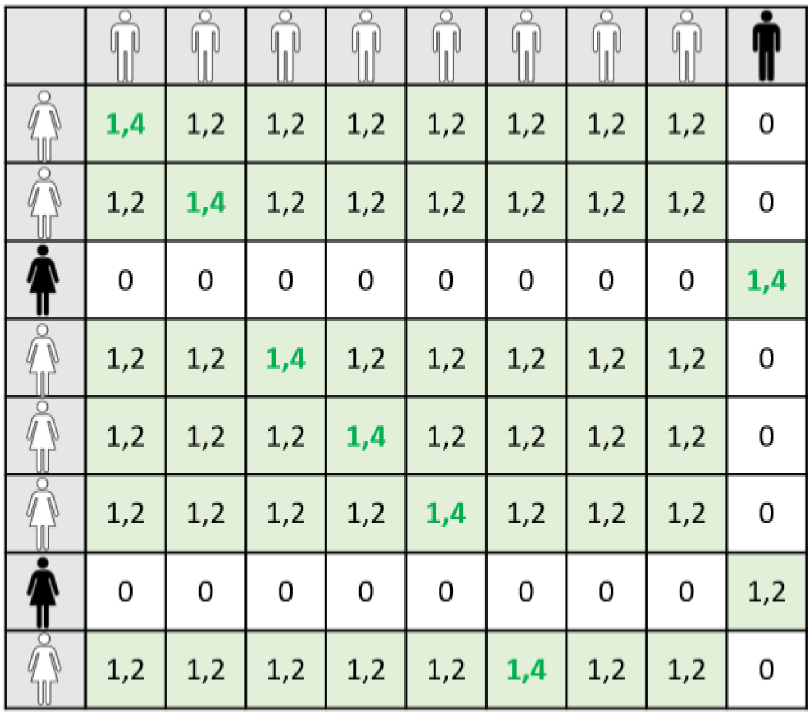

Figure S2. The simplified visualization of mutual assessment of candidate partners in case of the “assortative mating” scenario. The cells in the matrix are filled with the S score. Green cells – marriage is considered since the S score is higher than threshold values of both candidate agents. White cells – marriage is not considered since the S score is lower than threshold values of both candidate agents. Values in green are highest ones and the marriages are occurred.

The first step is to check if the candidates are brother and sister. For this, the values of the `fatherID` and `motherID` parameters of each candidate are compared, and if any of them coincide, the mutual evaluation is not conducted and the creation of such a couple becomes impossible.

If the candidates are not brothers and sisters, then the calculation of the `S` score begins according to the following rules:

1. If candidates have the same phenotype (both hearing or both deaf). then:

1.1. If both candidates are hearing. the values of the `WEIGHT_PHENO_H` parameters of both candidates are added to the `S` score (i.e. 2 × `WEIGHT_PHENO_H`).

1.2. If both candidates are deaf, the value 2 × `WEIGHT_PHENO_H` is added to the `S` score. Also added to the `S` score is the value `WEIGHT_SIGN_D`, if one of the candidates knows sign language, or 2 × `WEIGHT_SIGN_D`, if both candidates know sign language.

2. If candidates differ in phenotype (one is deaf, the other is hearing), then the sum (WEIGHT_SIGN_H + WEIGHT_SIGN_D) will be added to the S score only if both candidates know sign language.

After the S score is calculated, the program compares it with the sum of the socialPosition values obtained for each candidate. And if the S score exceeds the sum of the socialPosition thresholds, then the value of S is written in the cell of the matrix corresponding to the given pair of candidates.

Algorithm for generating offspring

If the marriage between the candidates took place, then the process of generating offspring begins.

First, the type of couple is determined based on the phenotypes of the partners (DD, DH or HH, where D is deaf, H is hearing). For couples of HH type, the value of the BIRTH_RATE_H parameter is used in further calculations; for couples of DD and DH types, the value of the BIRTH_RATE_D parameter is used. Next, the number of children for the current couple is determined using the beta distribution with the BETA_A and BETA_B parameters, the values of which are adjusted according to the BIRTH_RATE_D or BIRTH_RATE_H parameters, depending on the type of marriage. Based on the generated number of descendants, the program creates the corresponding number of new agents which are equally likely to be male or female.

The genotype of a new agent is formed based on the genotypes of the father and mother and is stored in the parameters mGenome (allele inherited from the mother) and fGenome (allele inherited from the father). The agent will be deaf for genetic reasons only if both alleles in the genotype are recessive, in which case the isDeaf parameter will have the value true. Whether the deafness of an agent is acquired is determined in the same way, as described above for the formation of the initial population. The knowsSignLanguage parameter (knowledge of sign language) is determined based on the family situation - if at least one deaf parent in the family knows sign language, then all children will automatically know it. The socialPosition parameter is determined as the arithmetic mean of the socialPosition parameters of the parents adjusted for some normally distributed value.

Chapter S3. Initial parameters

The following parameters are set in the model:

	**Parameter**	**Range of values**	**Description**
1	MAX_GENERATIONS	$1 \le x \le \infty$, $x \in N$	Number of generations
2	INIT_POP_SIZE_M	$1 \le x \le \infty$, $x \in N$	Initial size of male population
3	INIT_POP_SIZE_W	$1 \le x \le \infty$, $x \in N$	Initial size of female population
4	SPONTANEOUS_DEAF	$0 \le x \le 1$, $x \in R$	Proportion of agents with spontaneous deafness due to non-genetic reasons
5	DEAF_HOMOZYGOTES	$0 \le x \le 1$, $x \in R$	Proportion of the deaf in initial population
6	BIRTH_RATE_H	$0 \le x \le \infty$, $x \in N_0$	Average number of children of hearing agent
7	BIRTH_RATE_D	$0 \le x \le \infty$, $x \in N_0$	Average number of children of deaf agent
8	BETA_A. BETA_B	$x > 0$	Parameters for calculating beta distribution of birth rate
9	SOCIAL_MEAN_H	$0 \le x \le 1$, $x \in R$	Lower threshold of sum of parameter values of potential partner for *hearing* agent
10	SOCIAL_VAR_H	$0 \le x \le 1$, $x \in R$	Standard deviation of normal distribution for calculating SOCIAL_MEAN_H of specific *hearing* agent
11	SOCIAL_MEAN_D	$0 \le x \le 1$, $x \in R$	Lower threshold of sum of parameter values of potential partner for *deaf* agent
12	SOCIAL_VAR_D	$0 \le x \le 1$, $x \in R$	Standard deviation of normal distribution for calculating SOCIAL_MEAN_D of specific *deaf* agent
13	WEIGHT_PHENO_H	$0 \le x \le 1$, $x \in R$	Significance of phenotype match with potential partner for *hearing* agent
14	WEIGHT_SIGN_H	$0 \le x \le 1$, $x \in R$	Significance of sign language knowledge by potential partner for *hearing* agent
15	WEIGHT_PHENO_D	$0 \le x \le 1$, $x \in R$	Significance of phenotype match with potential partner for *deaf* agent
16	WEIGHT_SIGN_D	$0 \le x \le 1$, $x \in R$	Significance of sign language knowledge by potential partner for *deaf* agent

When generated, each agent receives the following set of parameters:

	Parameter	Range of values	Description
1	mGenome	{0, 1}<br>(1 – recessive allele, 0 - dominant)	Allele inherited from mother
2	fGenome	{0, 1}<br>(1 – recessive allele, 0 - dominant)	Allele inherited from father
3	isDeaf	true/false	Deafness of agent (true=deaf)
4	knowsSignLanguage	true/false	Knowledge of sign language
5	socialPosition	0≤x≤1, x∈R	Lower threshold of sum of parameter values of potential partner
6	fatherID		Agent father ID
7	motherID		Agent mother ID

Table S1. Initial simulation parameters for different scenarios.

No	Parameter	Scenario 1 “No deaf mating”	Scenario 2 “Assortative mating”	Scenario 3 “Random mating”	Verification USA data	Verification Yakut data
1	generations	21	21	21	20	7
2	init_pop_men	100000	100000	100000	100000	115000
3	init_pop_women	100000	100000	100000	100000	115000
4	candidate_pairs_mean	150	150	150	150	50
5	candidate_pairs_var	0	0	0	0	15
6	beta_ad	300.0f	300.0f	300.0f	1000.0f	100.0f
7	beta_bd	700.0f	700.0f	700.0f	100.0f	100.0f
8	spontaneous_deaf	0.0025f	0.0025f	0.0025f	0.00f	0.00f
9	deaf_homozygotes	0.0025f	0.0025f	0.0025f	0.0002f	0.00f
10	hear_homozygotes	0.8975f	0.8975f	0.8975f	0.9715f	1.0f
11	birth_rate_hearing	2.24f	2.24f	2.24f	2.24f	2.24
12	birth_rate_deaf	1.76f	1.76f	2.24f	2.016f	2.1
13	birth_rate_mixed	1.7f	1.7f	2.24f	2.128f	2.1
14	beta_a	2.0f	2.0f	2.0f	2.0f	2.0f
15	beta_b	5.0f	5.0f	5.0f	5.0f	5.0f
16	social_mean_h	0.3f	0.3f	0.3f	0.3f	0.55f
17	social_mean_d	0.3f	0.3f	0.3f	0.3f	0.55f
18	social_var_h	0.0f	0.0f	0.0f	0.0f	0.0f
19	social_var_d	0.0f	0.0f	0.0f	0.0f	0.0f
20	weight_phenotype_ hearing	0.4f	0.4f	0.4f	0.4f	0.6f
21	weight_phenotype_ deaf	0.0f	0.6f	0.0f	0.6f	0.3f
22	weight_sign_h	0.0f	0.4f	0.4f	0.4f	0.6f
23	weight_sign_d	0.0f	0.4f	0.4f	0.4f	0.3f
24	sign_lang_d	0.0f	1.0f	1.0f	1.0f	0.0f
25	sign_lang_h	0.0f	0.0f	1.0f	0.0f	0.0f
26	deaf_community_ model	0	1	0	1	0

Chapter S4. Verification and validation of the model

Table S2. Simulation results for validation scenario by USA data.

Years	Total Population (0.99 CI)	Deaf individuals (0.99 CI)	Allele frequency (0.99 CI)	Genotype frequency (0.99 CI)
0	200000.0 (±0.00)	40 (±0.00)	$1.435e-2$ ($\pm 0.000e-0$)	$2.000e-4$ ($\pm 0.000e-0$)
20	209643.4 (±0.37)	82.207 (±0.00)	$1.433e-2$ ($\pm 3.644e-6$)	$2.221e-4$ ($\pm 6.198e-10$)
40	219954.7 (±37.22)	52.134 (±0.62)	1.398e – 2 ($\pm 2.375e-5$)	$2.370e-4$ ($\pm 2.785e-6$)
60	230566.2 (±30.93)	65.677 (±1.08)	1.384e – 2 (±5.817e – 6)	$2.848e-4$ ($\pm 4.739e-6$)
80	241112.9 (±44.78)	76.19 (±0.37)	1.369e – 2 (±7.890e – 6)	$3.160e-4$ ($\pm 1.607e-6$)
100	252775.2 (±32.35)	88.931 (±0.22)	1.355e – 2 (±1.962e – 6)	$3.518e-4$ ($\pm 9.091e-7$)
120	264626.0 (±62.97)	98.005 (±1.21)	1.340e – 2 (±1.060e – 5)	$3.704e-4$ ($\pm 4.477e-6$)
140	276145.8 (±43.97)	112.332 (±1.18)	1.327e – 2 (±1.755e – 5)	$4.068e-4$ ($\pm 4.216e-6$)
160	289561.1 (±10.13)	121.13 (±2.08)	1.312e – 2 (±1.647e – 5)	$4.184e-4$ ($\pm 7.190e-6$)
180	303148.4 (±21.35)	117.908 (±0.25)	1.293e – 2 (±1.179e – 5)	$3.890e-4$ ($\pm 7.954e-7$)
200	316880.6 (±58.76)	120.677 (±2.98)	1.276e – 2 (±2.896e – 5)	$3.809e-4$ ($\pm 9.485e-6$)

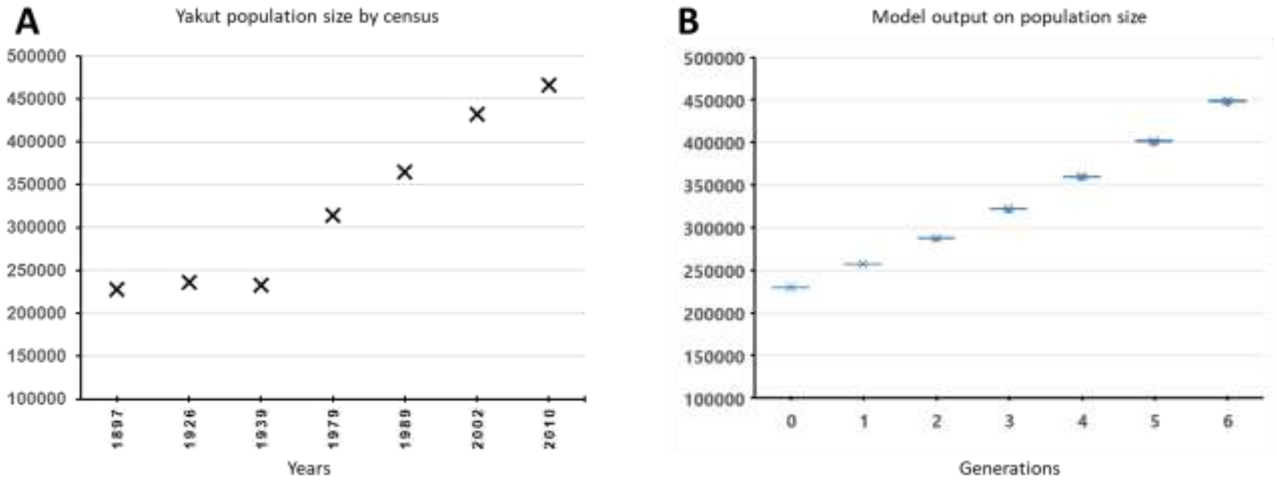

Figure S3. Model verification on Yakut population size dynamics. **A.** Yakut population number according to archive census data. **B.** Population size increase in the model outcome; Birth rate values was set to 2.24, which is a mean value for the number of children who survived to reproductive age in 1897 (2.18 per woman) and 2010 (2.31 per woman), according to archive census data.

Chapter S5. Modeling results

Table S3. Simulation results for scenario 1 “No deaf mating”

Years	Total Population (0.99 CI)	Deaf individuals (0.99 CI)	Allele frequency (0.99 CI)	Genotype frequency (0.99 CI)
0	200000.00 (±0.00)	999.31 (±0.37)	$5.250e-2$ ($\pm 0.000e-0$)	2.500e – 3 ($\pm 0.000e-0$)
20	222509.12 (±1.21)	1115.39 (±1.70)	$5.016e-2$ ($\pm 1.999e-5$)	2.520e – 3 (±1.406e – 6)
40	247652.42 (±12.30)	1184.19 (±2.51)	$4.778e-2$ ($\pm 2.399e-5$)	2.284e – 3 (±7.510e – 6)
60	275794.02 (±10.20)	1260.20 (±1.12)	$4.561e-2$ ($\pm 4.257e-5$)	2.074e – 3 (±4.793e – 6)
80	306397.56 (±29.84)	1347.15 (±0.71)	$4.361e-2$ ($\pm 2.091e-5$)	1.901e – 3 (±1.215e – 6)
100	340791.57 (±2.01)	1447.22 (±0.56)	$4.178e-2$ ($\pm 3.318e-5$)	1.748e – 3 (±1.445e – 6)
120	378288.16 (±12.24)	1550.50 (±0.46)	$4.011e-2$ ($\pm 1.029e-5$)	1.607e – 3 (±1.364e – 6)
140	421135.72 (±64.27)	1677.15 (±0.74)	$3.856e-2$ ($\pm 1.921e-6$)	1.483e – 3 (±1.117e – 6)
160	468272.88 (±66.19)	1816.89 (±0.40)	$3.713e-2$ ($\pm 1.886e-5$)	1.379e – 3 (±7.654e – 8)
180	522268.86 (±0.90)	1971.05 (±2.20)	$3.581e-2$ ($\pm 3.564e-7$)	1.280e – 3 (±2.613e – 6)
200	581591.03 (±52.74)	2149.63 (±1.18)	$3.457e-2$ ($\pm 3.245e-6$)	1.197e – 3 (±1.220e – 6)
220	648095.30 (±181.10)	2344.91 (±2.26)	$3.343e-2$ ($\pm 1.229e-5$)	1.116e – 3 (±1.648e – 6)
240	721688.52 (±69.48)	2557.64 (±3.53)	$3.235e-2$ ($\pm 7.757e-6$)	1.048e – 3 (±3.800e – 6)
260	804864.41 (±200.44)	2801.33 (±1.02)	$3.133e-2$ ($\pm 1.057e-5$)	9.807e – 4 (±3.750e – 7)
280	895747.78 (±94.95)	3065.79 (±3.04)	$3.038e-2$ ($\pm 1.405e-5$)	9.255e – 4 (±1.465e – 6)
300	998584.22 (±80.36)	3357.94 (±1.27)	$2.949e-2$ ($\pm 3.217e-5$)	8.681e – 4 (±9.537e – 7)
320	1111832.63 (±83.61)	3688.86 (±3.19)	$2.865e-2$ ($\pm 3.327e-5$)	8.204e – 4 (±3.414e – 6)
340	1239994.31 (±277.73)	4060.85 (±3.50)	$2.785e-2$ ($\pm 3.595e-5$)	7.753e – 4 (±1.003e – 6)
360	1382016.25 (±56.62)	4467.03 (±0.09)	$2.710e-2$ ($\pm 5.832e-6$)	7.344e – 4 (±1.413e – 6)
380	1541851.04 (±89.28)	4928.72 (±0.00)	$2.639e-2$ ($\pm 2.079e-6$)	6.984e – 4 (±3.613e – 7)
400	1721203.74 (±29.72)	5440.07 (±2.57)	$2.570e-2$ ($\pm 1.649e-6$)	6.612e – 4 (±2.462e – 7)

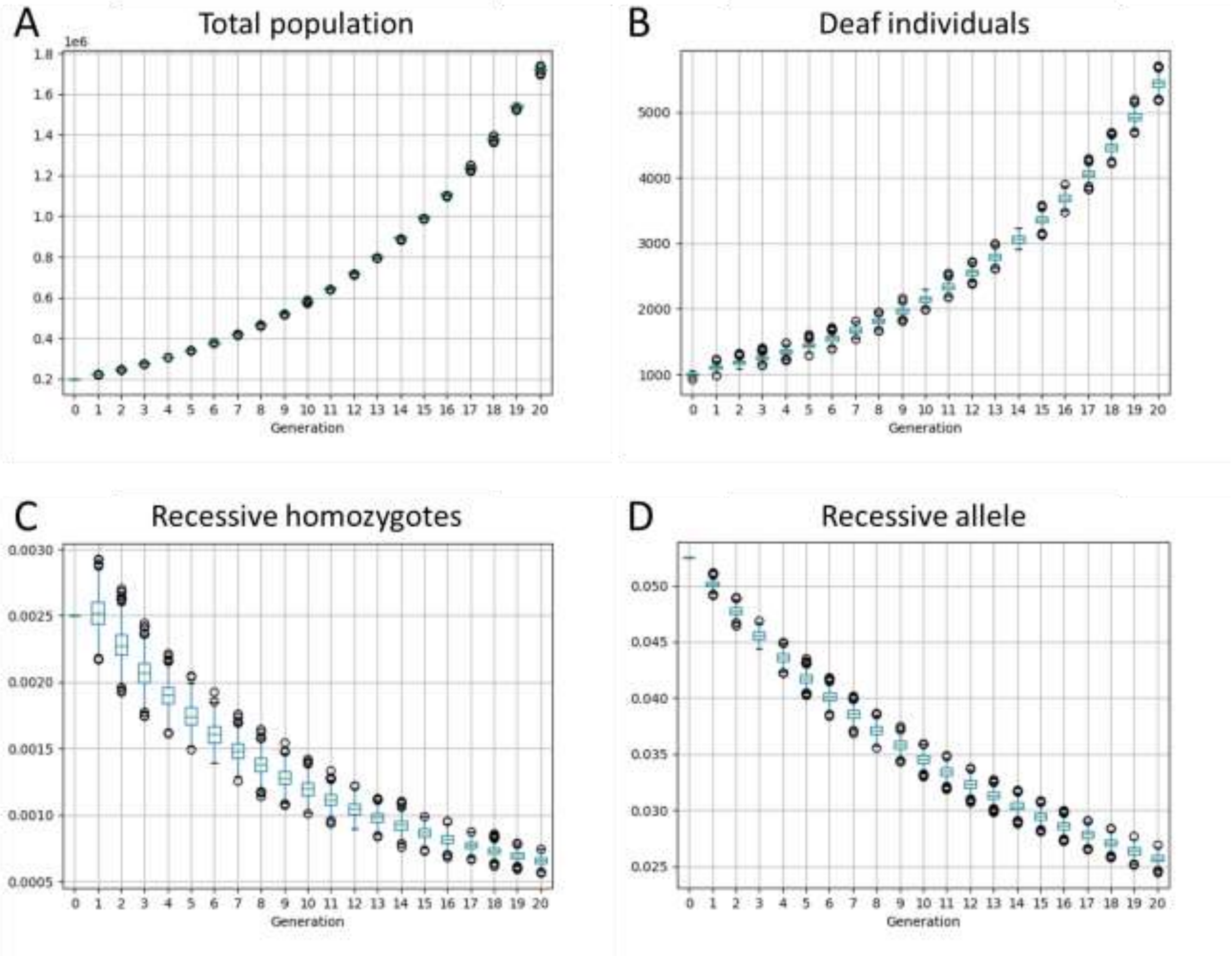

Figure S4. Simulation results for scenario 1 – “No deaf mating”. **A** – Total population size. **B** – The number of deaf individuals. **C** – Proportion of recessive mutant homozygotes in total population. **D** – Frequency of recessive mutant allele in total population.

Table S4. Simulation results for scenario 2 “Assortative mating”.

Years	Total Population (0.99 CI)	Deaf individuals (0.99 CI)	Allele frequency (0.99 CI)	Genotype frequency (0.99 CI)
0	200000.00 (±0.00)	997.60 (±1.55)	$5.250e-2$ ($\pm 0.000e-0$)	$2.500e-3$ ($\pm 0.000e-0$)
20	223317.53 (±2.65)	1333.18 (±0.86)	$5.184e-2$ ($\pm 8.915e-6$)	$3.471e-3$ ($\pm 3.508e-6$)
40	249219.39 (±12.91)	1480.78 (±3.95)	$5.112e-2$ ($\pm 1.884e-5$)	$3.454e-3$ ($\pm 1.358e-5$)
60	278625.21 (±36.37)	1742.06 (±6.92)	$5.044e-2$ ($\pm 7.671e-5$)	$3.767e-3$ ($\pm 1.999e-5$)
80	310574.08 (±44.92)	1968.69 (±1.43)	$4.969e-2$ ($\pm 9.412e-6$)	$3.852e-3$ ($\pm 6.251e-6$)
100	346489.86 (±22.28)	2187.96 (±7.09)	$4.890e-2$ ($\pm 5.241e-5$)	$3.827e-3$ ($\pm 2.380e-5$)
120	385614.24 (±49.36)	2425.23 (±6.52)	$4.813e-2$ ($\pm 1.425e-5$)	$3.800e-3$ ($\pm 1.354e-5$)
140	430365.08 (±111.96)	2641.69 (±3.01)	$4.736e-2$ ($\pm 8.854e-6$)	$3.647e-3$ ($\pm 5.206e-6$)
160	479762.15 (±135.99)	2873.08 (±0.33)	$4.663e-2$ ($\pm 2.330e-5$)	$3.500e-3$ ($\pm 4.403e-6$)
180	536425.92 (±34.70)	3128.89 (±0.94)	$4.594e-2$ ($\pm 8.626e-6$)	$3.342e-3$ ($\pm 7.888e-6$)
200	599440.47 (±272.34)	3411.63 (±3.10)	$4.526e-2$ ($\pm 3.504e-5$)	$3.201e-3$ ($\pm 2.598e-7$)
220	668929.41 (±128.45)	3712.52 (±0.53)	$4.462e-2$ ($\pm 1.430e-5$)	$3.058e-3$ ($\pm 1.096e-7$)
240	746558.94 (±127.92)	4083.75 (±3.58)	$4.399e-2$ ($\pm 4.819e-5$)	$2.980e-3$ ($\pm 1.600e-6$)
260	835138.41 (±50.54)	4492.56 (±0.29)	$4.338e-2$ ($\pm 1.482e-6$)	$2.885e-3$ ($\pm 2.700e-6$)
280	931372.24 (±5.21)	4931.38 (±0.73)	$4.281e-2$ ($\pm 4.076e-5$)	$2.805e-3$ ($\pm 5.954e-7$)
300	1040361.09 (±59.91)	5422.27 (±0.12)	$4.224e-2$ ($\pm 4.728e-6$)	$2.717e-3$ ($\pm 4.489e-6$)
320	1161796.74 (±269.17)	5955.77 (±5.29)	$4.169e-2$ ($\pm 4.197e-5$)	$2.633e-3$ ($\pm 2.393e-6$)
340	1297649.37 (±210.85)	6544.33 (±18.61)	$4.116e-2$ ($\pm 2.015e-5$)	$2.549e-3$ ($\pm 1.302e-5$)
360	1451073.88 (±672.37)	7196.66 (±10.39)	$4.064e-2$ ($\pm 9.184e-6$)	$2.466e-3$ ($\pm 1.739e-7$)
380	1621945.90 (±219.28)	7911.25 (±13.97)	$4.014e-2$ ($\pm 4.527e-5$)	$2.385e-3$ ($\pm 1.129e-5$)
400	1814625.89 (±142.06)	8719.41 (±15.80)	$3.964e-2$ ($\pm 4.643e-5$)	$2.311e-3$ ($\pm 7.075e-6$)

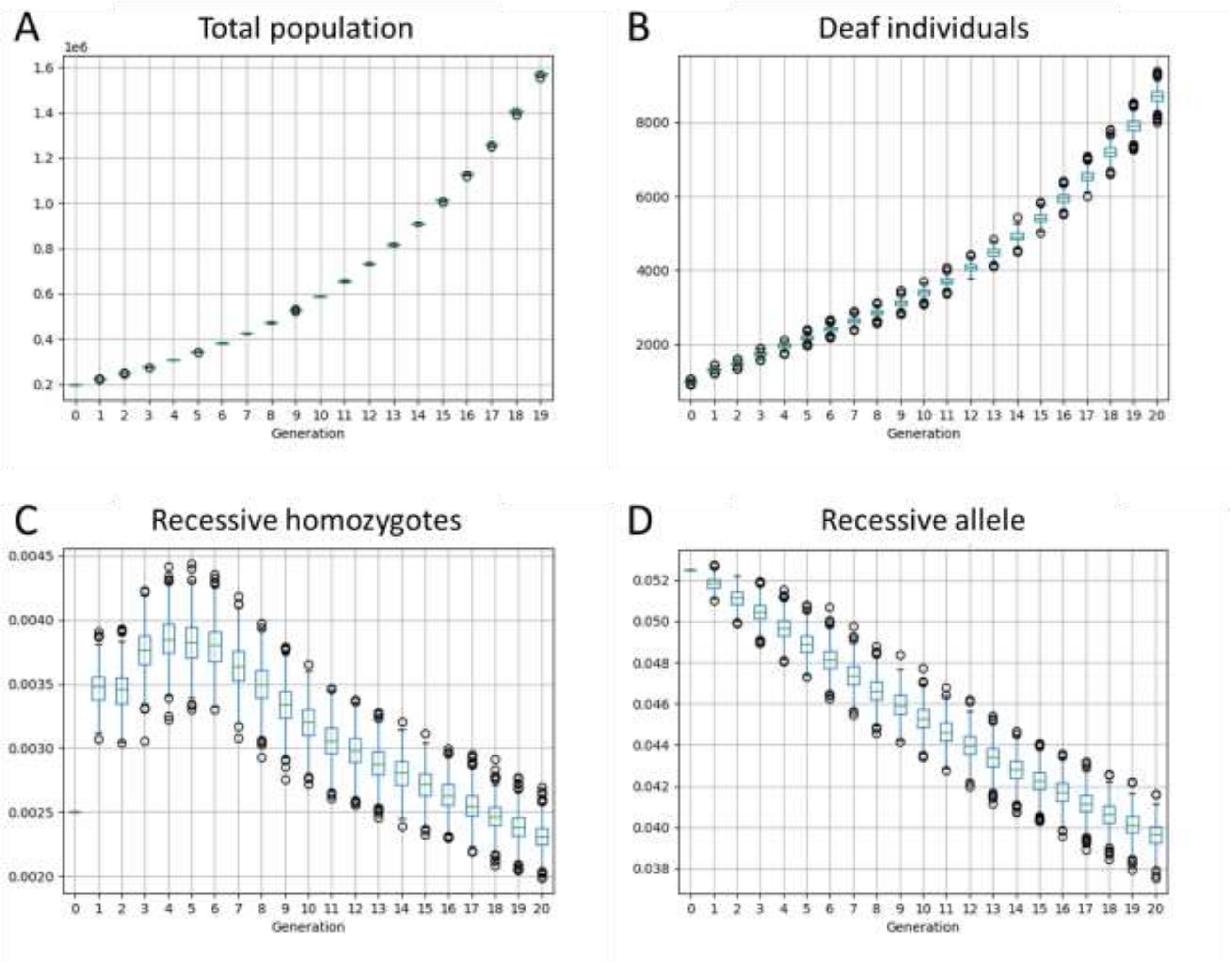

Figure S5. Simulation results for scenario 2 – "Assortative mating". **A** – Total population size. **B** – The number of deaf individuals. **C** – Proportion of recessive mutant homozygotes in total population. **D** – Frequency of recessive mutant allele in total population.

Table S5. Simulation results for scenario 3 “Random mating”.

Years	Total Population (0.99 CI)	Deaf individuals (0.99 CI)	Allele frequency (0.99 CI)	Genotype frequency (0.99 CI)
0	200000.00 (±0.00)	999.07 (±0.81)	$5.250e-2$ ($\pm0.000e-0$)	$2.500e-3$ ($\pm0.000e-0$)
20	223666.00 (±0.00)	1164.12 (±1.10)	$5.235e-2$ ($\pm3.013e-5$)	$2.716e-3$ ($\pm3.824e-6$)
40	250071.12 (±24.93)	1310.86 (±2.57)	$5.234e-2$ ($\pm3.926e-6$)	$2.747e-3$ ($\pm1.682e-6$)
60	280034.20 (±15.11)	1464.80 (±1.55)	$5.233e-2$ ($\pm4.252e-5$)	$2.741e-3$ ($\pm2.627e-6$)
80	312666.16 (±8.31)	1637.01 (±0.04)	$5.233e-2$ ($\pm3.870e-5$)	$2.738e-3$ ($\pm1.354e-6$)
100	349382.29 (±31.36)	1826.57 (±2.89)	$5.233e-2$ ($\pm1.649e-5$)	$2.735e-3$ ($\pm5.576e-6$)
120	389421.05 (±39.87)	2036.77 (±3.87)	$5.232e-2$ ($\pm5.820e-6$)	$2.740e-3$ ($\pm7.904e-6$)
140	435374.32 (±80.93)	2280.41 (±0.90)	$5.231e-2$ ($\pm1.902e-5$)	$2.738e-3$ ($\pm1.510e-6$)
160	486080.33 (±22.52)	2541.44 (±3.01)	$5.232e-2$ ($\pm2.097e-6$)	$2.733e-3$ ($\pm6.240e-6$)
180	544266.32 (±82.43)	2848.20 (±5.29)	$5.232e-2$ ($\pm1.752e-5$)	$2.741e-3$ ($\pm6.458e-6$)
200	609276.45 (±105.20)	3188.42 (±6.88)	$5.232e-2$ ($\pm4.871e-5$)	$2.737e-3$ ($\pm1.012e-5$)
220	680797.93 (±61.70)	3556.74 (±0.00)	$5.232e-2$ ($\pm5.992e-5$)	$2.737e-3$ ($\pm6.201e-6$)
240	761091.75 (±63.01)	3978.40 (±1.95)	$5.233e-2$ ($\pm1.898e-6$)	$2.738e-3$ ($\pm4.625e-6$)
260	852284.26 (±128.70)	4455.51 (±2.36)	$5.231e-2$ ($\pm2.160e-5$)	$2.740e-3$ ($\pm1.709e-6$)
280	951729.71 (±208.89)	4979.58 (±16.49)	$5.232e-2$ ($\pm1.248e-4$)	$2.738e-3$ ($\pm1.271e-5$)
300	1064259.76 (±250.88)	5560.88 (±1.02)	$5.232e-2$ ($\pm2.801e-5$)	$2.733e-3$ ($\pm2.959e-6$)
320	1190192.44 (±432.20)	6226.55 (±2.61)	$5.233e-2$ ($\pm3.262e-6$)	$2.740e-3$ ($\pm3.315e-7$)
340	1331127.28 (±370.70)	6968.70 (±14.91)	$5.233e-2$ ($\pm6.648e-5$)	$2.743e-3$ ($\pm8.701e-6$)
360	1490730.70 (±92.00)	7805.32 (±22.52)	$5.233e-2$ ($\pm1.573e-4$)	$2.740e-3$ ($\pm1.435e-5$)
380	1668175.88 (±204.94)	8726.21 (±17.51)	$5.233e-2$ ($\pm9.518e-5$)	$2.738e-3$ ($\pm8.467e-6$)
400	1868655.58 (±345.94)	9776.90 (±0.16)	$5.233e-2$ ($\pm3.965e-5$)	$2.737e-3$ ($\pm2.145e-6$)

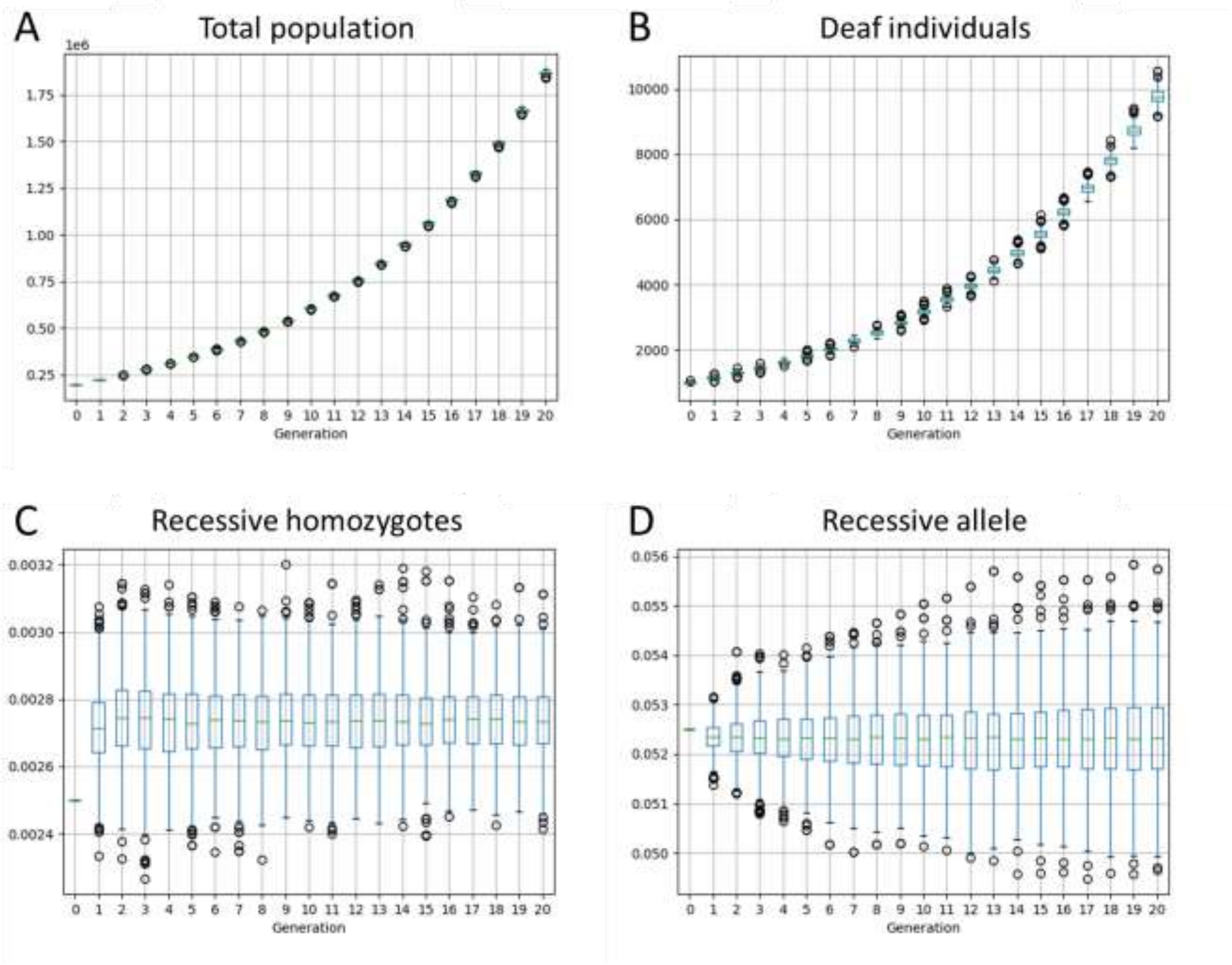

Figure S6. Simulation results for scenario 3 “Random mating”. **A** – Total population size. **B** – The number of deaf individuals. **C** – Proportion of recessive mutant homozygotes in total population. **D** – Frequency of recessive mutant allele in total population.
